# Lectures on the Theory and Practice of Midwifery

**Published:** 1844-04

**Authors:** 


Art. V.—*Lectures on the Theory and Practice of Midwifery.* By R. Lee, m.d. f.r.s. With numerous Wood-engravings.—*London*, 1844. 8vo, pp. 560.

Dr. Lee's recent work on Clinical Midwifery, showed him to have had unusually great opportunities of acquiring a practical knowledge of the obstetric art. The usefulness of that work, however, was somewhat diminished by the absence of those explicit directions, with reference to difficult points of practice, which are so essential for the guidance of the inexperienced. That want, however, is now fully supplied by the publication of these lectures, which form a most important addition to our obstetric literature. The first fourteen lectures are occupied with anatomical and physiological details, in which Dr. Lee has the great advantage of having been himself a diligent and successful investigator of those subjects which he describes. The next six lectures treat of the symptoms of pregnancy, and the disorders attending it; and the succeeding twenty-six are occupied with the description of natural and preternatural labours, and the treatment appropriate to each. In these lectures especially, Dr. Lee's lucid descriptions, his explicit directions, and the high tone of feeling which he everywhere shows, and earnestly inculcates, deserve the highest praise. The last seven lectures, from the thirty-eighth to the forty-fourth, are occupied with a notice of some of the chief diseases of the puerperal state—including puerperal fever, and phlegmasia dolens, with reference to which time seems to have confirmed the opinions expressed by the author in his Essays on some of the most important Diseases of Women.

The woodcuts which illustrate the work are well chosen and well executed, and the elaborate statistical tables which it contains are extremely valuable. It will, we doubt not, extend Dr. Lee's already high reputation, and be found extremely useful by practitioners generally as well as by students. They can have no safer or more enlightened guide. In the next edition, the index, which is now very imperfect, should be made much more complete; as facility of reference is a point of great importance in all works intended as manuals for students and men in practice.

